# Usual Populations, Unusual Individuals: Insights into the Behavior and Management of Asian Elephants in Fragmented Landscapes

**DOI:** 10.1371/journal.pone.0042571

**Published:** 2012-08-15

**Authors:** Nishant M. Srinivasaiah, Vijay D. Anand, Srinivas Vaidyanathan, Anindya Sinha

**Affiliations:** 1 Postgraduate Program in Wildlife Biology and Conservation, National Centre for Biological Sciences, Tata Institute of Fundamental Research, GKVK Campus, Bangalore, India; 2 A Rocha India, Bangalore, India; 3 Foundation for Ecological Research, Advocacy and Learning, Appavounagar, Vazhakulam, Pondicherry, India; 4 School of Natural Sciences and Engineering, National Institute of Advanced Studies, Indian Institute of Science Campus, Bangalore, India; 5 Nature Conservation Foundation, Mysore, India; University of California, Berkeley, United States of America

## Abstract

**Background:**

A dearth in understanding the behavior of Asian elephants (*Elephas maximus*) at the scale of populations and individuals has left important management issues, particularly related to human-elephant conflict (HEC), unresolved. Evaluation of differences in behavior and decision-making among individual elephants across groups in response to changing local ecological settings is essential to fill this gap in knowledge and to improve our approaches towards the management and conservation of elephants.

**Methodology/Principal Findings:**

We hypothesized certain behavioral decisions that would be made by Asian elephants as reflected in their residence time and movement rates, time-activity budgets, social interactions and group dynamics in response to resource availability and human disturbance in their habitat. This study is based on 200 h of behavioral observations on 60 individually identified elephants and a 184-km^2^ grid-based survey of their natural and anthropogenic habitats within and outside the Bannerghatta National Park, southern India during the dry season. At a general population level, the behavioral decisions appeared to be guided by the gender, age and group-type of the elephants. At the individual level, the observed variation could be explained only by the idiosyncratic behaviors of individuals and that of their associating conspecific individuals. Recursive partitioning classification trees for residence time of individual elephants indicated that the primary decisions were taken by individuals, independently of their above-mentioned biological and ecological attributes.

**Conclusions/Significance:**

Decision-making by Asian elephants thus appears to be determined at two levels, that of the population and, more importantly, the individual. Models based on decision-making by individual elephants have the potential to predict conflict in fragmented landscapes that, in turn, could aid in mitigating HEC. Thus, we must target individuals, in addition to populations, in our efforts to manage and conserve this threatened species, particularly in human-dominated landscapes.

## Introduction

An important, often-neglected, aspect of behavioral ecology concerns the ability of animal populations and individuals to respond to changes in their immediate environment, both in the long- and the short- term. Systematic changes in behavioral patterns in response to predictable variation in the environment such as resource availability, including those that may occur seasonally, could be innate and selected for [1]. For example, behavioral responses encompassed by varying time-activity budgets, spatial and temporal preferences for certain habitats or the avoidance of areas in response to certain ecological parameters have been documented extensively [1], [2]. Such responses may often be determined by relatively more biologically determined factors such as the gender, age or the typical social organization of the species concerned. What might be more problematic for animals, however, are the demands placed on them by short-term, unpredictable ecological changes in their environment, a classic example being of animal populations that largely occur in increasingly human-dominated landscapes. This might be more challenging for large-bodied mammalian species such as the elephant that lives in complex societies but which may be able to cope with rapid environmental changes with their learning capacities and sophisticated cognitive decision-making abilities [3].

The availability and distribution of resources seem to primarily influence the occurrence of elephants in a particular region [3]–[7]. Although this species does not have any natural predator [3], humans and anthropogenic disturbances have, over time, emerged as major threats to these mammals, occasionally threatening their very survival. Demographic changes brought about by illegal hunting may demand that elephants rapidly adapt behaviorally to such drastic ecological changes [8]–[10], but which have rarely been documented. Moreover, what remain virtually unknown are the behavioral differences displayed by groups and individuals in response to these change. Such a dearth in our understanding of the behavioral decisions made by elephants, influenced both by their biology and their ecology, has left important management issues, particularly related to human-elephant conflict (HEC), unresolved and rendered ineffective several mitigation measures that have been adopted to reduce conflict in anthropogenic habitats.

Our current knowledge of elephants show that they are highly polygynous and sexually dimorphic animals [11]. They are also long-lived with well-defined growth phases, punctuated importantly by puberty. In males, body size dictates dominance and reproductive success [11]–[14]. Older and larger male elephants have been observed to come into *musth* more often and be in that state for longer durations of time than do younger males [3], [14], [15]. These males are also known to control the *musth* and regulate the social behavior of younger bulls [15]. Bulls in *musth* are known to have higher reproductive success than those not in *musth* [14]. In the African elephants, females, are known to form a fission-fusion social structure with a strong social hierarchy among herds and clans, which are herds that associate with one another [16]–[17]. Recent pioneering studies on Asian elephants by de Silva *et al* show that, unlike their African counterparts, female societies in Asian elephants do not maintain coherent core groups and exhibit significantly less social bonding at the population level. They suggest, however, that strong social networking does occur at different levels of association and that this unique multilevel social organization, which does not follow the classical hierarchical structure, must be recognized among Asian elephants [18]. Social dominance and rank hierarchy among herds may, in turn, determine their distribution in an area and the use of certain habitats [17], [19], [20]. In females, dominance has been suggested to be an offshoot of the age of the individual. The oldest female acts as the matriarch and influences the behavior of other individuals in the herd [3], [20]. Studies in Africa have shown the existence of exclusive bull areas and the differential seasonal use of different areas by males and females depending on their resource requirements and the need to avoid human-related threats [14].

Behavioral patterns displayed by elephants are a result of their decision-making processes and could be influenced by their innate biology as well as the prevailing ecology. For example, a number of studies, both in Asia and Africa, have attributed crop-raiding behavior mostly to adult males [3], [21], [22]. In the Asian elephant, males, are typically born in a herd, reach puberty at about the age of 10 to 15 years, form loose associations with other males or live solitarily, and associate with herds thereafter only for mating [3]. These decisions seem to be primarily governed by the requirements of a certain age or a growth phase. A detailed assessment of the biological and ecological factors that are pivotal to decision-making in elephants have, however been rarely explored.

A notable exception is the recent study by Chiyo *et al.* that shows how life-history characteristics and inter-individual interactions influence decision-making by the male African elephant [22]. They argue that simplistic models of exposure of an animal to crops do not fully explain its crop-raiding behavior and establish the influence of life-history traits such as age and energy requirements, as well as cultural traits such as social learning, on their raiding behavior. This study, however, explores the behavior of individual male elephants in all-male groups alone and does not take into account the presumably important influence of mixed age-sex herds on such decision-making processes. More importantly, this study does not consider the often-idiosyncratic crop-raiding decisions made by individual elephants, independently of their biological attributes and socioecological environments, but which, we think, are crucial determinants of behavioral decisions, including crop-raiding, made by Asian elephants. It is, therefore, crucial that we comprehensively identify the factors driving behavioral decisions by the Asian elephant not only to understand its biology better but also to develop more effective management strategies for it; this is especially true in the light of its increasing conflict with humans, which has emerged as possibly its most important conservation threat in the 21^st^ century. It is perhaps essential that we distinguish between decision-making processes that are relatively more influenced by innate biological features of the species such as gender or age and those that are more idiosyncratic, being determined at the level of the individual. Management strategies would then have to be designed to address problems posed by particular individuals rather than for the population at large.

In this study, we assessed the variation in decision-making by individuals of an Asian elephant population, residing in a fragmented human-dominated landscape, in response to varying levels of resource availability (forage, water and shade) as well as human disturbance. Residence time and movement rates, time-activity budgets, social interactions and group dynamics were used as quantitative measures of such decision-making processes. We first explored the influence of innate biological variables such as gender, age and group type on these behavioral responses. We further investigated the possibility of individual idiosyncrasies impacting these decisions and manifest as variability in residence time of individually identified elephants in a particular habitat and area. This was considered important as elephants could conceivably have strategies to acquire resources and avoid threats that are unique to them as individuals.

We hypothesized the following behavioral manifestations of the decisions that could possibly be made by our study elephants in their highly fragmented habitat.

At a general population level, Elephants would prefer resource-rich areas and avoid highly disturbed areas; The time-activity budget of individual elephants would be influenced by human disturbance and could lead to an increase in vigilance-related behaviors with a concomitant decrease in the time spent in foraging-related activities; Individuals would associate in smaller groups, reduce social interactions and increase movement rates in highly disturbed areas; Elephants would differ in their levels of occurrence in disturbed areas and in their utilization of resources in relation to their gender, age and group type; At an individual level, The residence time in different kinds of habitats, characterized by varying levels of resource availability and human disturbance, could vary across individuals idiosyncratically, independent of their gender, age and group type.

## Results

### General population-level behavioral pattern

#### Elephants Occur Mostly in Resource-Rich and Undisturbed Areas

**Figure 1:**
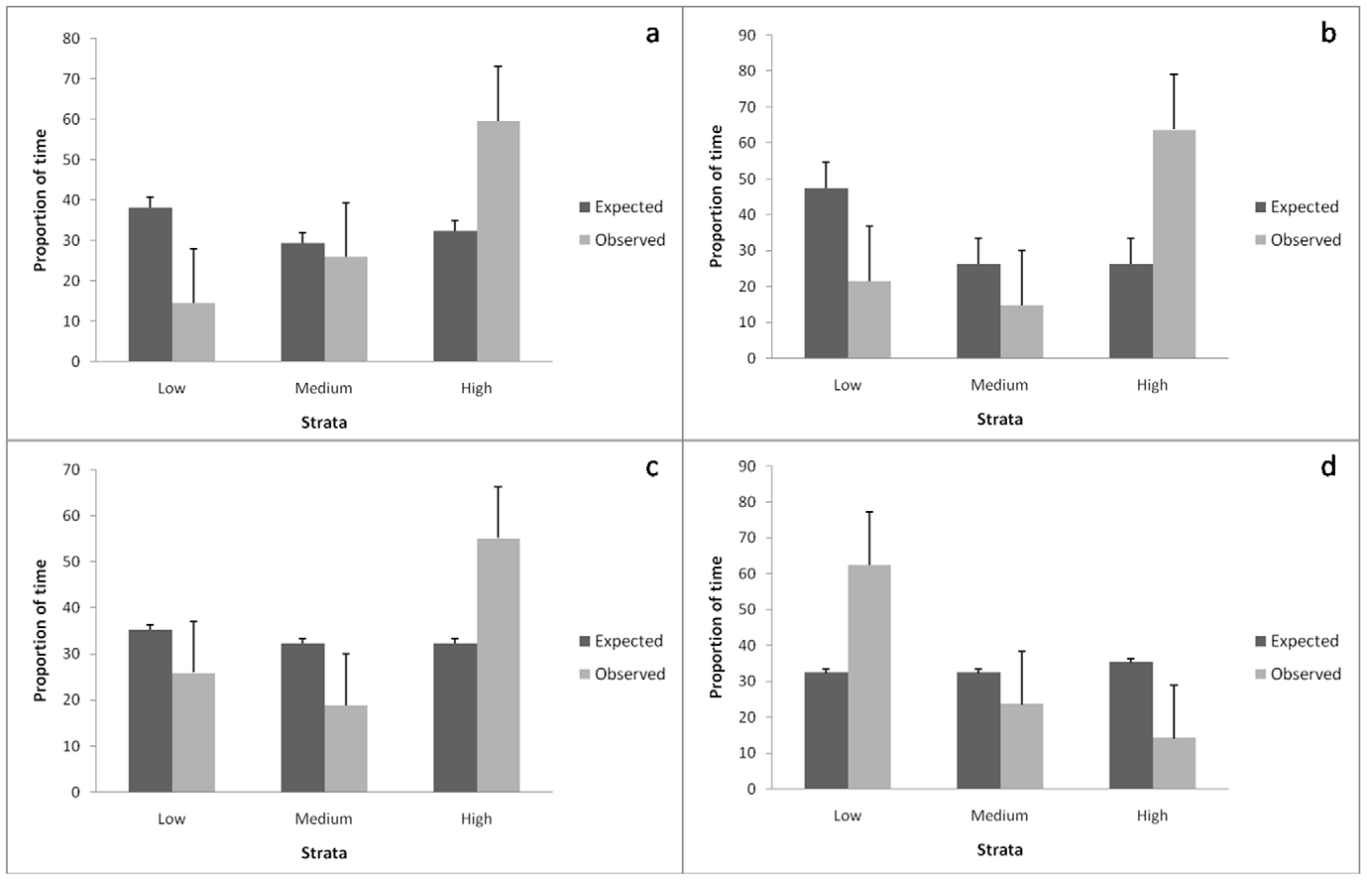
Expected and Observed Residence Time of elephants in the different strata. Low-, medium- and high levels of (a) forage-, (b) water-, (c) shade availability and (d) human disturbance.

We quantified the probability of elephants to occur with different residence times in strata that were independently classified as high, medium or low with reference to their resource (forage, water or shade) availability and human disturbance (Figure 1). We used Pearson's correlation to assess the multicollinearity amongst the measured variables. The variables forage and shade alone were found to be significantly correlated (r = 0.48, p<0.001) while the other variables were not. Our field observations on elephants, however, indicated that vegetation cover during the day and the cover of darkness at night played a key role in influencing decision-making in elephants. We, therefore, treated shade as a surrogate for cover availability and have, accordingly, presented the results of the G-tests of goodness of fit and independence for shade. We hypothesized that elephants would occur more in strata where forage, water and shade were relatively more abundant and less in strata with high human imprint. Our results show that indeed elephants did have significantly higher Observed Residence Times in strata that had relatively greater availability of forage (G-test of goodness of fit, G = 270.33, df = 2, p<0.001), water (G = 403.50, p<0.001) and shade (G = 168.13, p<0.001), and those with relatively low human disturbance (G = 231.99, p<0.001) than what would be expected if Observed Residence Times depended on the area available in each kind of stratum independent of resource availability and human disturbance in these strata (Figure 1).

#### Increasing Human Disturbance Reduces Effective Feeding Time, Alters Movement and Increases Time Spent Standing Alert

**Figure 2:**
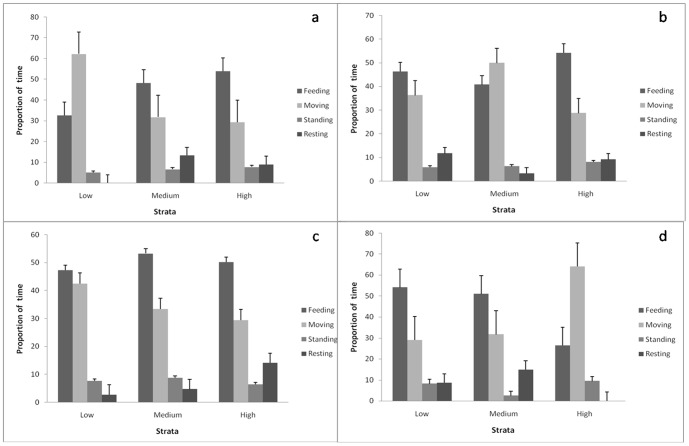
Proportion of time spent in feeding, moving, standing and resting in different strata. Low-, medium- and high levels of (a) forage-, (b) water- and (c) shade availability and (d) human disturbance.

An examination of the overall time-activity budget of the study elephants showed that they spent a majority of their time in foraging-related behaviors, including feeding (45.63%) and moving (30.48%). These behaviors and resting, measured as proportion time spent in these behaviors, increased in habitats with increasing forage and shade, but decreased in those with high human disturbance (Figure 2). These behaviors, however, did not differ significantly with water availability. The proportion of time spent feeding increased with increasing forage (G-test of goodness of fit, G = 40.57, df = 6, p<0.001) and decreased with increasing disturbance (G = 54.20, p<0.001). It should be noted that the proportion of time spent in feeding was highest during morning (57.14%) than during the other times of the day including the night. Moving, decreased with increasing forage (G = 40.57, p<0.001) and shade (G = 13.15, p = 0.041). It, however, increased with increasing disturbance (G = 54.20, p<0.001). Moving occurred most significantly during the night (49.12%). The elephants increased the proportion of time standing alert with increase in human disturbance (G = 40.57, p<0.001) but also with increasing forage (G = 54.20, p<0.001) and water (G = 13.58, p = 0.035). Resting was virtually absent in highly disturbed areas (G = 40.57, p<0.001) and increased significantly with increasing shade (G = 13.15, p = 0.041).

#### Elephants Associate in Smaller Groups, Reduce Social Interactions and Increase Movement Rates in Highly Disturbed Areas

**Figure 3:**
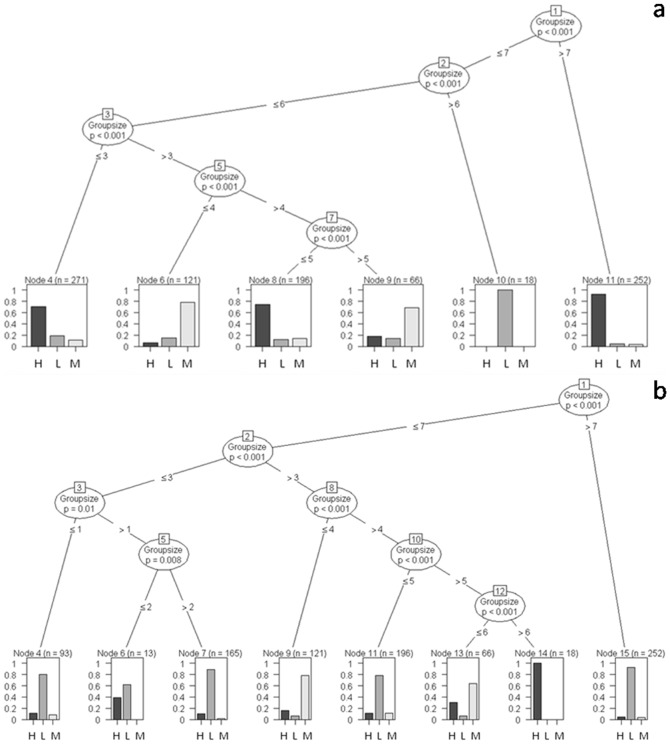
Classification trees showing the partitioning of elephant group size in the different strata. L: low-; M: medium- and H: high (a) forage availability and (b) human disturbance. The y-axis of each graph indicates the proportion of groups observed in the different strata. For the trees depicting the influence of water- and shade availability, see Figure S1.

The group size of elephants in the study area varied from a minimum of 1 to a maximum of 12, with a mean (± SE) of 6.50 (±0.06) individuals (Figure 3 and Figure S1). The mean (± SE, range) group size in high-disturbance – low-resource, medium-disturbance – medium-resource and low-disturbance – high-resource areas were 5.67 (±0.13, 1–8), 4.89 (±0.07, 1–8) and 7.09 (±0.08, 1–12) respectively. Group sizes greater than seven were not observed in high-disturbance – low-resource areas. Elephant group size significantly differed across varying levels (high, medium and low) of forage (G-test of independence, G = 710.56, df = 18, p<0.001), water (G = 620.88, p<0.001), shade (G = 439.44, p<0.001) and human disturbance (G = 844.88, p<0.001) than what would be expected of these habitats by chance alone.

The study elephants displayed increasing levels of bunching behavior, with lowered Inter-Individual Distance values, with increasing levels of human disturbance (Wilcoxon rank sum test with continuity correction, W = 465.50, N = 113, p<0.01). They vocalized audibly more frequently when in less-disturbed areas than they did in those with high human disturbance (G test of goodness of fit, G = 16.11, df = 2, p<0.001).

**Figure 4:**
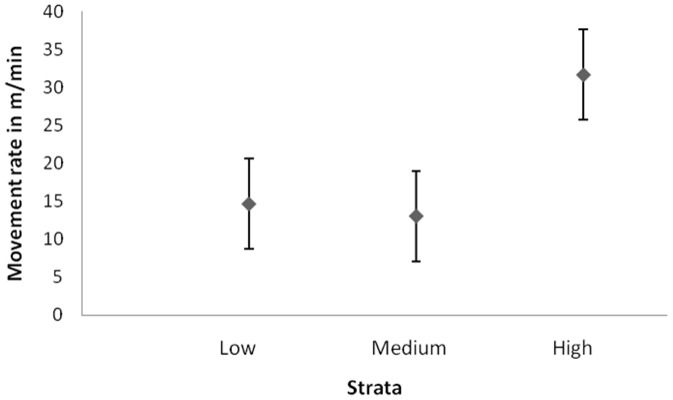
Movement rate (mean ± SE) of elephants in different strata of human disturbance.

We hypothesized that elephants would move rapidly across highly disturbed areas in order to minimize their exposure to threat by humans. Our study elephants moved at a rate four times higher across high-disturbance areas as compared to that in low-disturbance areas (Figure 4). The movement rate was least in high-forage (8.97 m/min) and high-shade (8.90 m/min) areas and highest in areas with high human disturbance (31.65 m/min) and medium forage (37.06 m/min). The rate of movement also varied significantly in strata with different levels of forage (G-test of goodness of fit = 24.30, df = 2, p<0.001), shade (G = 10.02, p = 0.006) and disturbance (G = 11.09, p = 0.003). It did not, however, vary with differing water availability (G = 0.03, p = 0.98).

#### Elephants Vary in Their Residence Time in Different Habitats Depending on Their Gender, Age and Group-type

**Figure 5:**
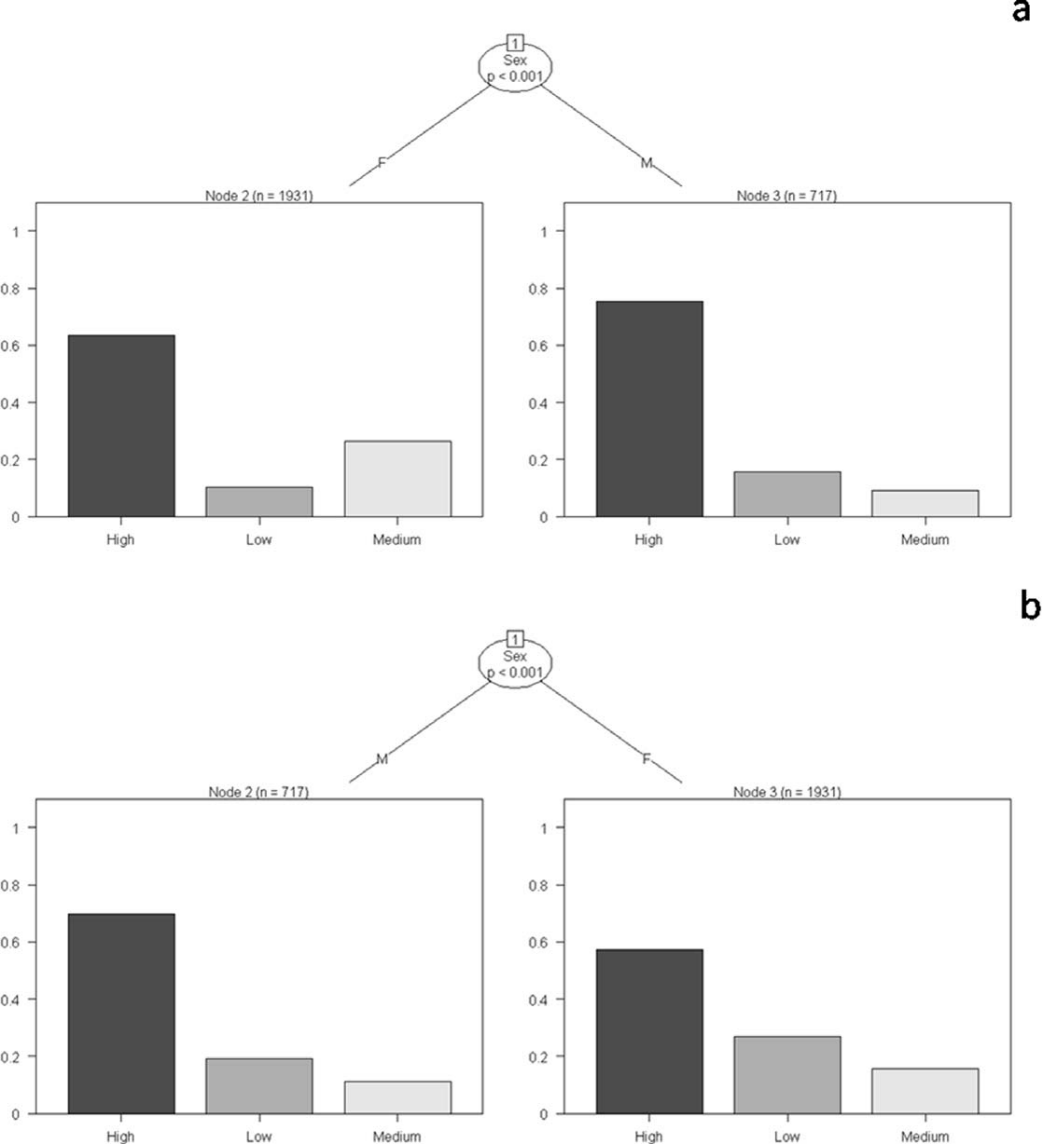
Classification trees for residence time of female and male elephants in the different strata. (a) Forage- and (b) water availability. The y-axis of each graph indicates the proportion of groups observed in the different strata. For the trees depicting the influence of shade availability and human disturbance, see Figure S2.

Both male and female elephants occurred primarily in high-resource – low-disturbance areas (Figure 5 and Figure S2). The genders, however, differed in their residence time across different strata with varying levels of forage (G-test of independence, G = 112.18, df = 2, p<0.001), water (G = 35.45, p<0.001) and human disturbance (G = 87.25, p<0.001). No difference, however, could be detected with varying shade (G = 6.48, p>0.05). Female elephants thus occurred in medium-forage – medium-disturbance areas at levels higher than that of males (Figure 5; G = 112.18, p<0.001). Male elephants, in contrast, occurred at significantly higher levels in high-forage areas (G = 112.18, p<0.001) and at significantly lower levels in low-disturbance areas (G = 87.25, p<0.001).

**Figure 6:**
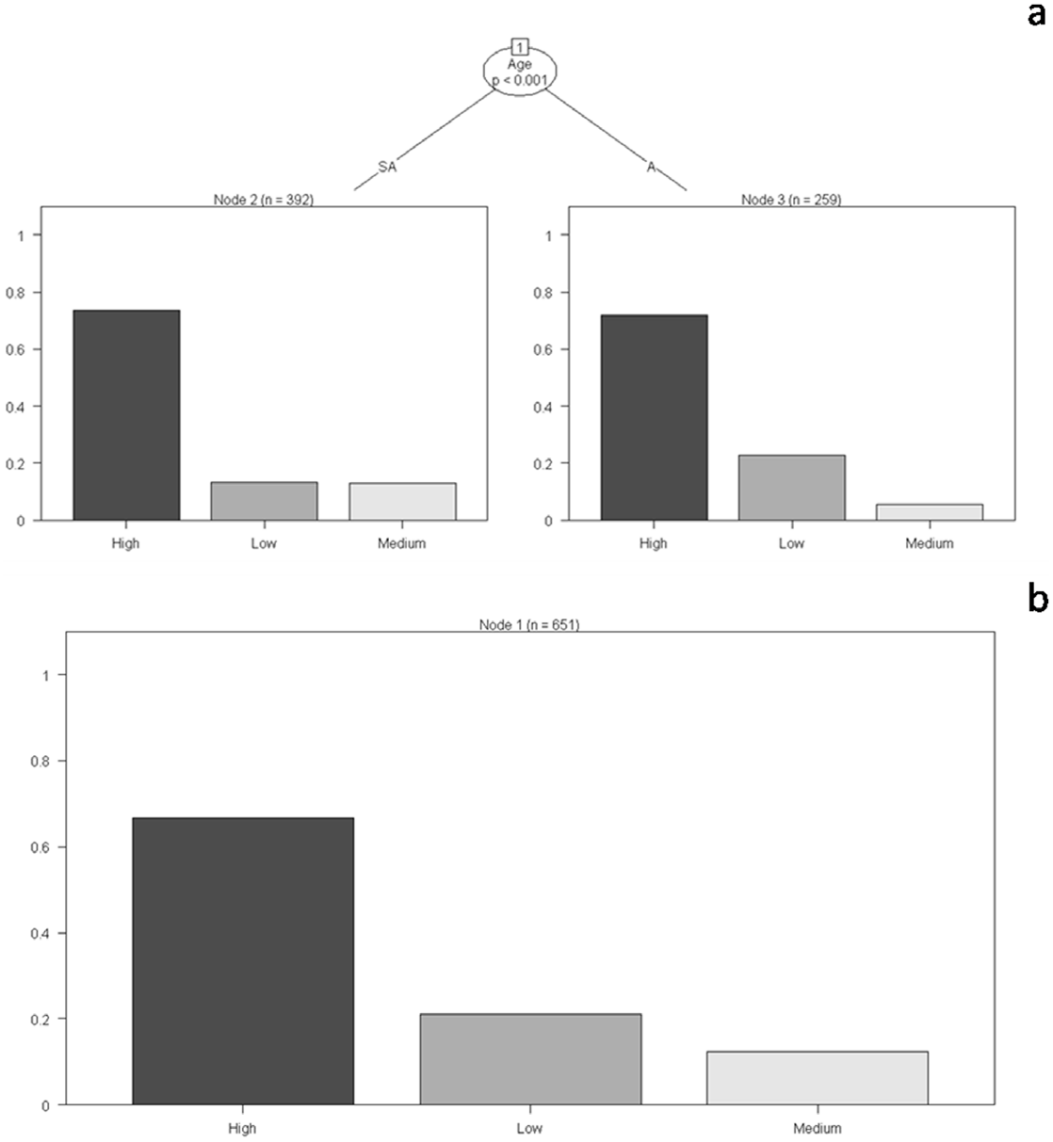
Classification trees for residence time of adult and subadult male elephants in the different strata. (a) Forage- and (b) water availability. The y-axis of each graph indicates the proportion of groups observed in the different strata. For the trees depicting the influence of water- and shade availability, see Figure S3. For the trees depicting the influence of forage-, water-, shade availability and human disturbance on residence time of adult and subadult female elephants, see Figure S4.

We observed no difference between the different age classes of elephants across the sexes (adult, subadult, juvenile and calf) in their residence time in habitats with differing levels of forage, water and human disturbance. Their occurrence, however, differed with varying levels of shade (G = 6.43, p = 0.04). When only males were considered, adult elephants occurred in high-forage – low-disturbance areas at levels higher than did subadults. The latter age class, on the other hand, occurred in medium-forage – medium-disturbance areas at levels higher than the former. Adult and subadult males differed in their residence time in strata with different forage (Figure 6 and Figure S3; G = 85.69, p<0.001), shade (G = 54.89, p<0.001) and disturbance (G = 44.25, p<0.001). Adult and subadult female elephants also showed differences in their occurrence in habitats with varying levels of forage (Figure S4; G = 16.94, p<0.001) and disturbance (G = 17.90, p<0.001).

**Figure 7:**
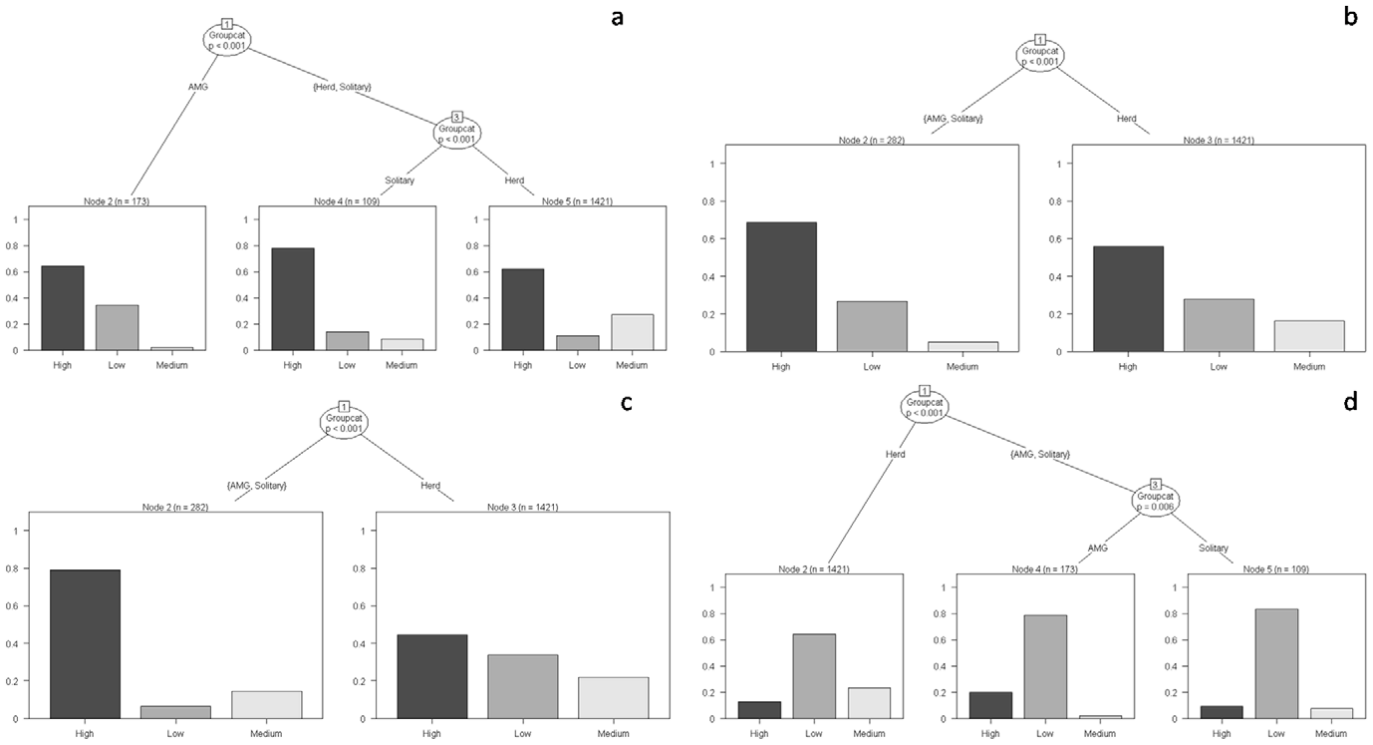
Classification trees for residence time of adult and subadult male elephants in the different strata as a function of group types. (a) Forage-, (b) water- and (c) shade availability and (d) human disturbance. The y-axis of each graph indicates the proportion of groups observed in the different strata.

When individual adult and subadult male elephants associated to form All Male Groups (AMG), they showed a higher propensity of occurrence in high-disturbance areas. Solitary elephants, in contrast, exhibited the least propensity to occur in such areas, followed by Herds (Figure 7). There were significant differences in the occurrence of these group types in different habitats as a function of forage availability (G = 539.36, df = 4, p<0.001) and human disturbance (G = 400.85, p<0.001). Solitary individuals and AMG differed from Herds in their residence time with varying levels of water (G = 90.81, p<0.001) and shade (G = 41.754, p<0.001). No difference was, however, observed between Solitary elephants and AMG across differing availability of water (Node 2, p>0.05) and shade (Node 2, p>0.05).

### Specific individual-level behavioral patterns

Recursive partitioning classification trees constructed for residence time of individual elephants indicated that the primary decisions made by our study elephants occurred at the level of the individual independent of their biological and ecological attributes. This variability in behavioral decisions could, at best, only be attributed to individual idiosyncrasies. Some of these decisions made by identified individuals, however, changed when they associated with certain conspecific individuals.

**Figure 8:**
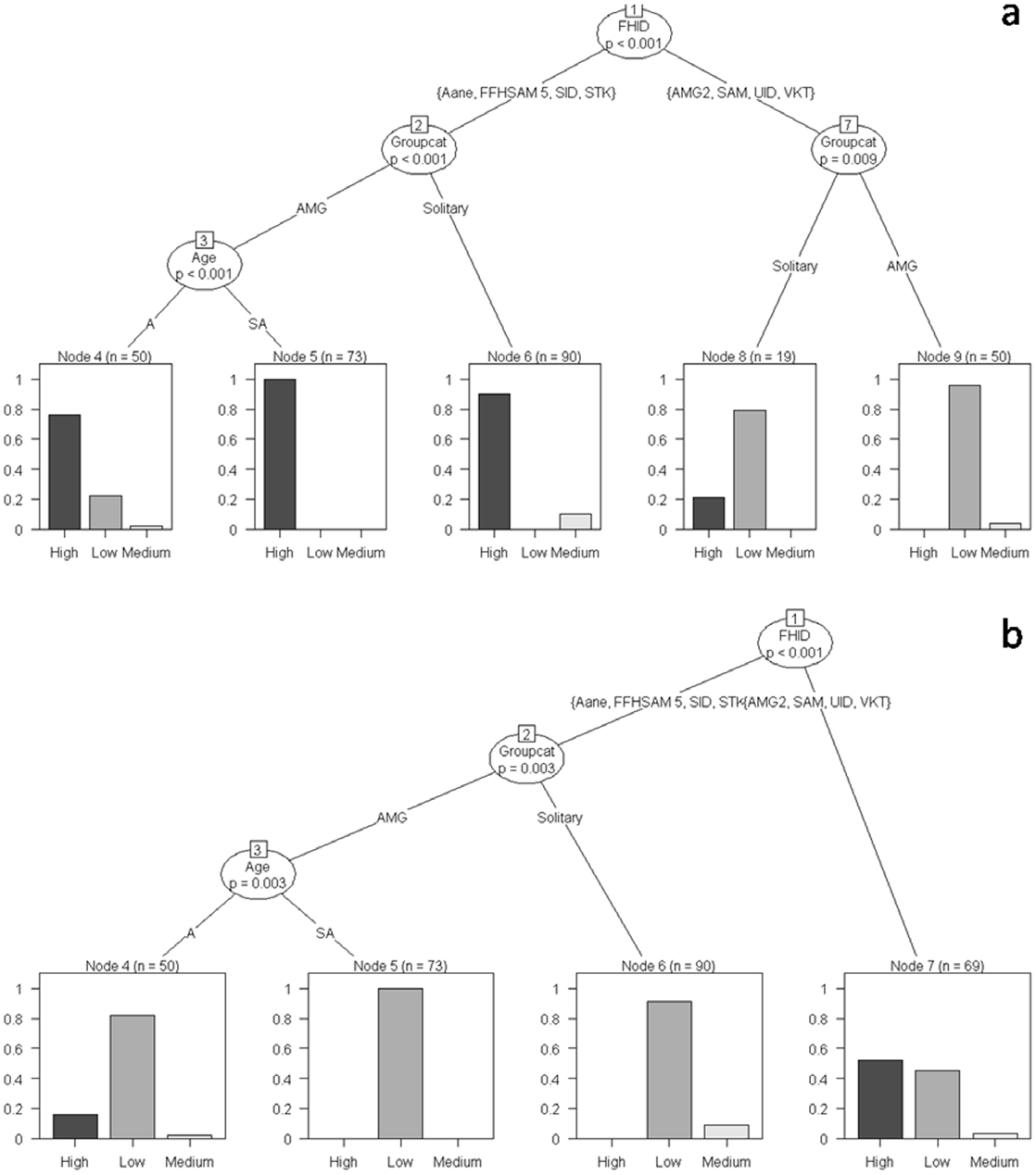
Classification trees of the probability of individual adult and subadult male elephants occurring in the different strata. (a) Forage availability and (b) human disturbance. The y-axis of each graph indicates the proportion of groups observed in the different strata. For the trees depicting the influence of water- and shade availability, see Figure S5.

The total number of scans on identified individual adult and subadult males (>10 years) ranged from 5 to 119 with a mean (± SE) of 32.60 (±11.83) and that on identified herds from 4 to 560 with a mean (± SE) of 238 (±61.10). We have, however, chosen individual adult and subadult males with a minimum number of 35 scans and 8.75 h of observation on each individual and herds with 419 scans and 104.75 h of observation on each herd for further analysis. As an example, let us consider a particular classification tree shown in Figure 8. This tree depicts decision-making among our study adult and subadult male elephants, either when Solitary or in AMG, in response to resource availability and human disturbance measured on ordinal scales. The adult male VKT displayed a high propensity to occur in high-disturbance areas (Node 1) as compared to the other males and his behavior did not change even when he associated independently with other conspecific individuals. Two other males, SID and Stalker, in contrast, clearly differed in their residence time in different habitats as a function of the group in which they occurred (Node 2, p = 0.004). For example, when these males were solitary, they preferentially occurred in low-disturbance areas (Node 3); when in AMG, however, they increased their propensity to occur in high-disturbance areas (G-test of independence, G = 17.11, df = 2, p<0.001). When decision-making by SID and Stalker was considered in relation to a different ecological variable, forage availability, however, they showed an increased propensity to spend significantly higher residence time in high-forage areas when compared to VKT (Node1). When in AMG, however, SID showed a tendency to spend more time in low-forage areas as compared to Stalker (G = 14.30, df = 2, p<0.001). Figure S5 lists the classification trees that reveal variability in residence time displayed by individual adult and subadult male elephants under water- and shade availability.

**Figure 9:**
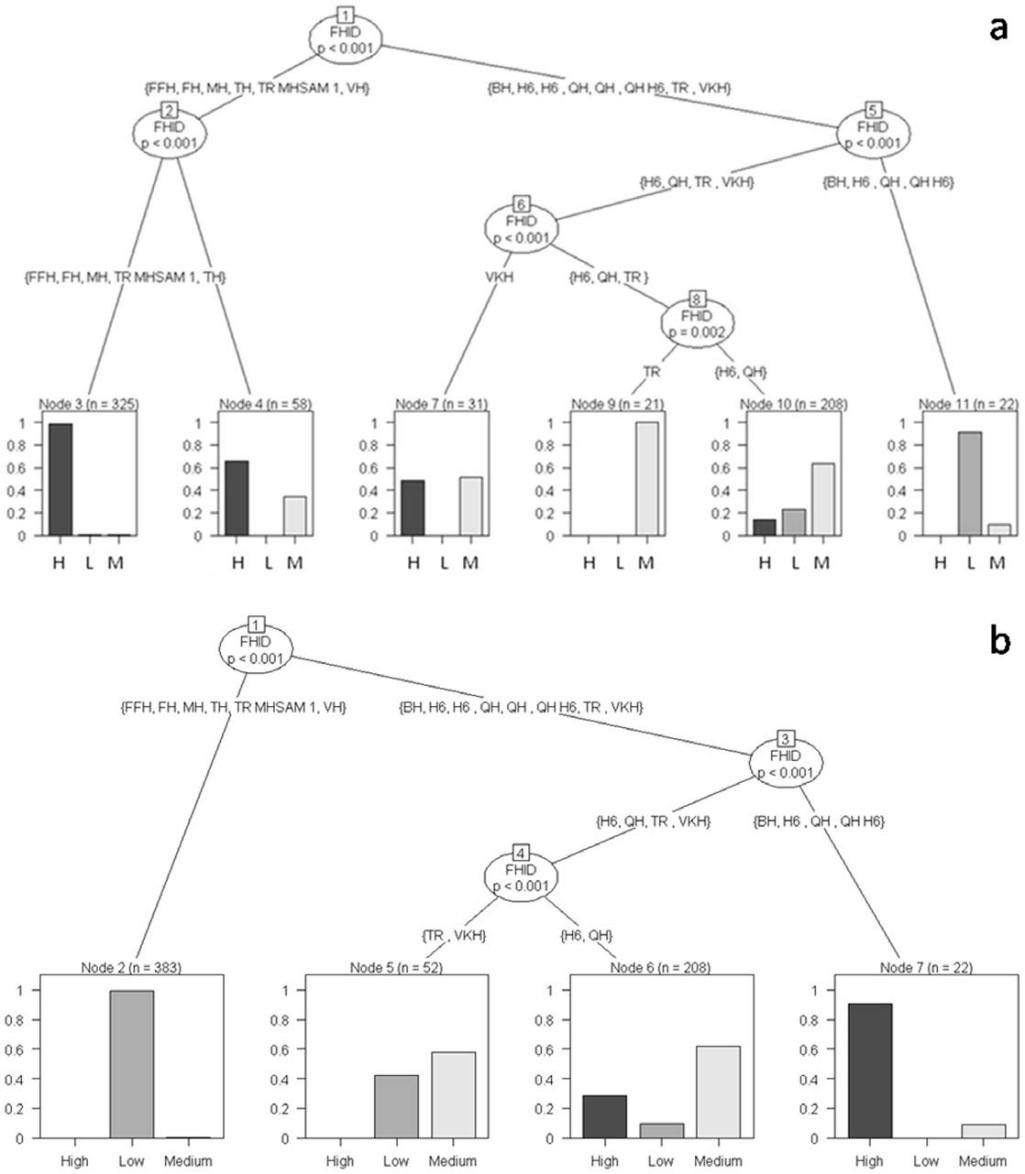
Classification trees of the probability of an individual herd occurring in the different strata. L: low-; M: medium- and H: high (a) forage availability and (b) human disturbance. The y-axis of each graph indicates the proportion of groups observed in the different strata. For the trees depicting the influence of water- and shade availability, see Figure S6.

Individuality was also observed across Herds (Figure 9). Herd QH, for example, displayed the highest propensity of occurrence in low-forage – high-disturbance areas (Nodes 3 and 2 respectively) as compared to the Herds FFH and MH, which had highest residence times in high-forage – low-disturbance areas (Node 5 and 3 respectively). Figure S6 shows the classification trees that reveal variability in residence time displayed by individual Herds under water- and shade availability.

## Discussion

The habitat of the Asian elephant is increasingly becoming fragmented due to anthropogenic influences across the distribution range of the species [23]. Many protected areas in the Indian subcontinent that harbor a high density of elephants nevertheless record intense human-elephant conflict in their fringe villages if not within the protected area itself [24]. The Bannerghatta National Park in southern India is one such elephant landscape that faces high levels of conflict, particularly related to crop raiding by elephants. Any attempt to manage and conserve this species is thus crucially dependent on our understanding of the behavioral decisions made by elephants in such disturbed ecological settings. The current study is possibly the first to extensively investigate the variation in decision-making by Asian elephants as reflected in their residence time, time-activity budgets, social interactions and grouping patterns under varying levels of resource availablity and human disturbance. It appears that such decisions are critically influenced, on the one hand, by biological and socioecological factors at the population level, such as gender, age and grouping patterns, and, on the other, by individual idiosyncracies.

The elephants in the study area appear to selectively respond to locally dynamic ecological situations by preferring to reside in resource-rich, but low human-disturbance areas, a pattern that has been observed earlier in both Asian and African elephants [4]–[6], [25]–[32]. Such risk-avoidance behavior may have aided in the long-term survival of elephants in fragmented habitats by way of their spatial and temporal separation from humans thus keeping conflict levels low [8], [19], [20]. Increasing human disturbance, by way of degradation of the quality and quantity of vegetation within the protected area and disrupting free access to and the use of crucial resources by the elephants within the Park, however, may lead to escalated conflicts in the near future and may, in turn, affect stress-related mortality patterns in such populations.

The rapidly-changing land-use patterns that characterize the neighborhood of the Bannerghatta National Park and the seasonal variability in resource availability within the Park, especially during the dry season, not only alters the time-activity budgets of elephants but also leads to significant changes in their use of the habitat, depending on their gender, age and group-types.

A reduction in foraging-related activities with a corresponding increase in vigilance behaviors was, thus, observed among our study elephants in areas with high human disturbance. The proportion of time spent feeding, for example, reduced from 54.08% in low human-disturbance areas to 26.44% in high human-disturbance areas. Resting ceased in highly human-disturbed areas, with the proportion of time spent moving increasing to 64.07% in these areas from 29.07% in areas relatively less disturbed by people, a finding that reflects in Asian elephants suggestions of similar patterns in their African counterparts [19], [33]. Elephants in and around the study area are constantly persecuted by local communities who depend on these habitats for their resources, especially farmers whose cropfields are raided by the elephants. The time spent standing alert also increased significantly in disturbed areas; the elephants usually froze as a first reaction to the close proximity of humans while they waited for the perceived threat to pass. Increased movement away from people was observed only when targeted efforts were made to drive them away, either from cropfields or from areas where livestock had been taken to graze.

We used recursive partitioning classification trees, as an exploratory analysis tool, to statistically test and visualize the differences observed in behavioral decision-making by elephants in relation to low-, medium- and high levels of resource availability and human disturbance. The observed variation in residence time of elephants, as a function of more general level attributes such as group size, gender, age and group-type, as well as at the individual level were all assessed in these strata using classification trees. Such an analysis has possibly never been used before to unravel behavioral decision-making in elephants and can potentially be a powerful tool to explain elephant occurrence and habitat use in different environments.

Group size in elephants is known to increase with a concomitant increase in resource availability and reduce when resources become limiting [3]. The group size of Asian elephants in dry habitats has been found to usually range between 5 and 10 individuals [3]. The elephants in the study area, all identified individuals, actively reduced their group size not only in relation to reduction in resource availability but also to increasing human disturbance, all observed differences in group size being statistically significant. Associating in relatively larger numbers in highly human-disturbed areas could lead to greater detectability and hence, crop-raiding in smaller units could be a more prudent strategy for these elephants. Support for such a hypothesis comes from our own personal observations when we were able to detect larger groups of elephants with relatively greater ease, even in darkness. A second possibility could be that elephants prefer to raid cropfields in family units alone, especially during times of resource limitation, as direct benefits would then only accrue to highly related kin groups.

Our observations on the bunching behavior of the study elephants, associated with a reduction in inter-individual distance in highly human-disturbed areas has been observed in other studies too, both in the Asian and African elephant [8]. The demonstrated increase in stress in elephants, possibly associated with a heightened sensitivity to human disturbance and a lack of resources, could have other significant effects on their behavior and physiology [33]. Our study elephants, for example, significantly reduced their audible vocalizations when in highly human-disturbed areas. This was particularly true when they raided cropfields or moved through areas with rampant cattle grazing or firewood collection. Actively reducing loud vocalizations could be a learnt behavior, as we observed that such vocalizations, even though rare, elicited frequent antagonistic responses, usually first from dogs and then from people; such learning could well be reinforced in an area such as the Bannerghatta National Park which has had a long history of human-elephant conflict [34]. This study, however, did not attempt to establish whether these elephants shifted from audible to infrasound communication during these times. Another behavioral adaptation that we observed was a significant increase in the rate of movement displayed by the elephants especially when travelling through areas of high human disturbance. Such a potential adaptation to anthropogenic threats has been documented earlier when elephants moved faster across forested tracts that were highly human-disturbed but connected resource-rich patches [4], [19], [33].

Gender and age are two important attributes that influence decision-making by elephants. Earlier studies on both Asian and African elephants have described differential patterns of habitat use by the two sexes although there have been very few investigations on the influence of age on such patterns [3]. What needs to be explored further, however, are the underlying motivations that drive individuals of different age-sex categories to selectively utilize areas of differing resource availability and human disturbance.

The high observed propensity of herds to occur in medium-forage – medium-disturbance areas and actively avoid high-forage – low-disturbance areas, which are usually frequented by adult males, for example, could be a behavioral response of females to avoid such males, possibly due to an inter-sexual dominance hierarchy that we believe exists in this species just as it does in the African elephant [20], [35], [36]. Such a dominance hierarchy usually manifests itself when adult males seek out herds with receptive females and display assertive behaviors towards its members, especially receptive females and young males [3]. Thus, the use of medium-forage – medium-disturbance areas by adult females could be a strategy [19] to ensure resources not only for themselves but for the rest of the herd, especially calves and juveniles, while reducing risk of encountering humans as well as certain male elephants. Female elephants, accompanied by calves and juveniles, also showed a high propensity to occur in areas with high water availability.

In polygynous species, the physical and physiological condition of males determines their positions in the dominance hierarchy and usually influences the outcome of inter-specific competition [12], [37], [38]. Male elephants need to build body mass, come into the energetically demanding state of *musth* and seize opportunities to mate. Male elephants, thus, as is typical of most polygynous species, track both forage and females [13], [14]. The occurrence of adult male elephants in high-forage – low-disturbance areas could therefore be explained as a need to maximize their energy gain and simultaneously minimize anthropogenic threats in order to improve their fitness. Subadult males, moving out of their natal herds and attempting to acquire social status [3], [20], [39], may also prefer high-forage – low-disturbance strata areas in order to build up body mass and to associate with adult males. Their presence in medium-forage – medium-disturbance areas at levels higher than that of adult males could possibly be expected as subadult individuals occasionally continue to associate with their natal herds.

The tendency of individual elephants to associate with one another to form transient or stable same-sex or mixed-sex groups, or to remain solitary may be an important component of their life-history strategies. Although the influence of all-male associations on crop raiding by African elephants has only very recently been explored [22], the factors motivating individual Asian elephants to form specific associations in different socioecological environments needs to be thoroughly investigated. All-male groups (AMG), formed by the association of adult and subadult males and which have been noted earlier in both Asian and African elephants [3], [12], could act as transient cooperative groups in which individuals could learn survival skills from one another [12], [22]. These associations could also represent genetically related kin groups, as has been demonstrated in African elephants [37]. These groups most often occurred in the high-disturbance – low-forage strata than in any other kind of forage- or disturbance regime during our study. Although such a choice of habitat could appear counter-intuitive, individual males in AMG tended to be more exploratory and often raided cropfields that required them to traverse high-disturbance tracts. They were perhaps able to withstand impending threats due to their close association with one another without the encumbrance of dependent young, as faced by herds.

Herds, in contrast to AMG or solitary males, typically occurred in medium-forage – medium-disturbance regimes, as has been discussed above. There were, however, a few notable exceptions. Herd QH differed from the others by showing a high tendency to frequent highly disturbed areas. Although a single study each on both Asian and African elephants have suggested that herds lower in the social hierarchy within a population are forced to reside in areas with relatively higher human disturbance [17], [19], such unusual herd behavior could also depend on two other factors, the first, as discussed above, being group size. A second, hitherto unexplored factor, could be the behavioral propensities of individual elephants comprising a particular herd. Understanding the behavioral profiles and social relationships of individual elephants within herds could serve to explain certain patterns of herd distribution and behavior that remain inexplicable, especially in fragmented human-dominated landscapes.

One of the most significant findings of this study, we believe, is that the occurrence of an individual elephant in a particular stratum could be a consequence of its age or grouping pattern, neither of which is static over time. We have thus demonstrated the ability of individual elephants to change behavioral strategies depending on whether they were solitary or in different group-types. What must be recognized, therefore, is that the adoption of particular strategies usually reflect the different demands placed on individuals depending on their life-history stage. Understanding this behavioral variability of individual elephants within a particular population to the extent that their behavioral strategies can be predicted is a challenging exercise but one that must be attempted if we are to develop viable management approaches to mitigate human-elephant conflict (HEC) or, more specifically, engage with conflict animals that occur in high-disturbance areas, comprising mostly of crop fields and human habitations. This becomes particularly crucial if we plan to implement any management strategy such as capture, translocation or any other action that additionally interferes with and disrupts the social fabric of individual elephants.

In conclusion, this study clearly shows that elephants behaviorally select high-resource areas and avoid anthropogenically disturbed areas. Several behavioral activities of elephants, especially foraging, movement and social interactions, are affected by increasing disturbance levels. Thus, the management of an area for elephants must aim at reducing these disturbances. At a population level, elephants differ in their residence times within particular areas, depending on their gender, age and grouping patterns. This clearly indicates differential needs and strategies among elephants within a population. Individual level studies are thus vital in order to identify the needs of particular animals and correctly predict their behavior. This also suggests that the management of an area should be dynamic and that we need to develop predictive models that would allow us to cater to the needs of a particular elephant population and also to the demands of each individual within it. Such an approach can immensly aid managers to take on-gound well-informed decisions in order to manage conflict, especially when dealing with highly endangered temperamental animals such as elephants.

The Asian elephant is clearly a species that displays extensive social behavior, complex cognitive abilities and sophisticated decision-making processes. Given these capabilities, both at the population- and individual level, a thorough understanding of elephant behaviour is absolutely crucial for any science-based management action that aims to conserve this increasingly endangered species.

## Materials and Methods

### Study area

**Figure 10:**
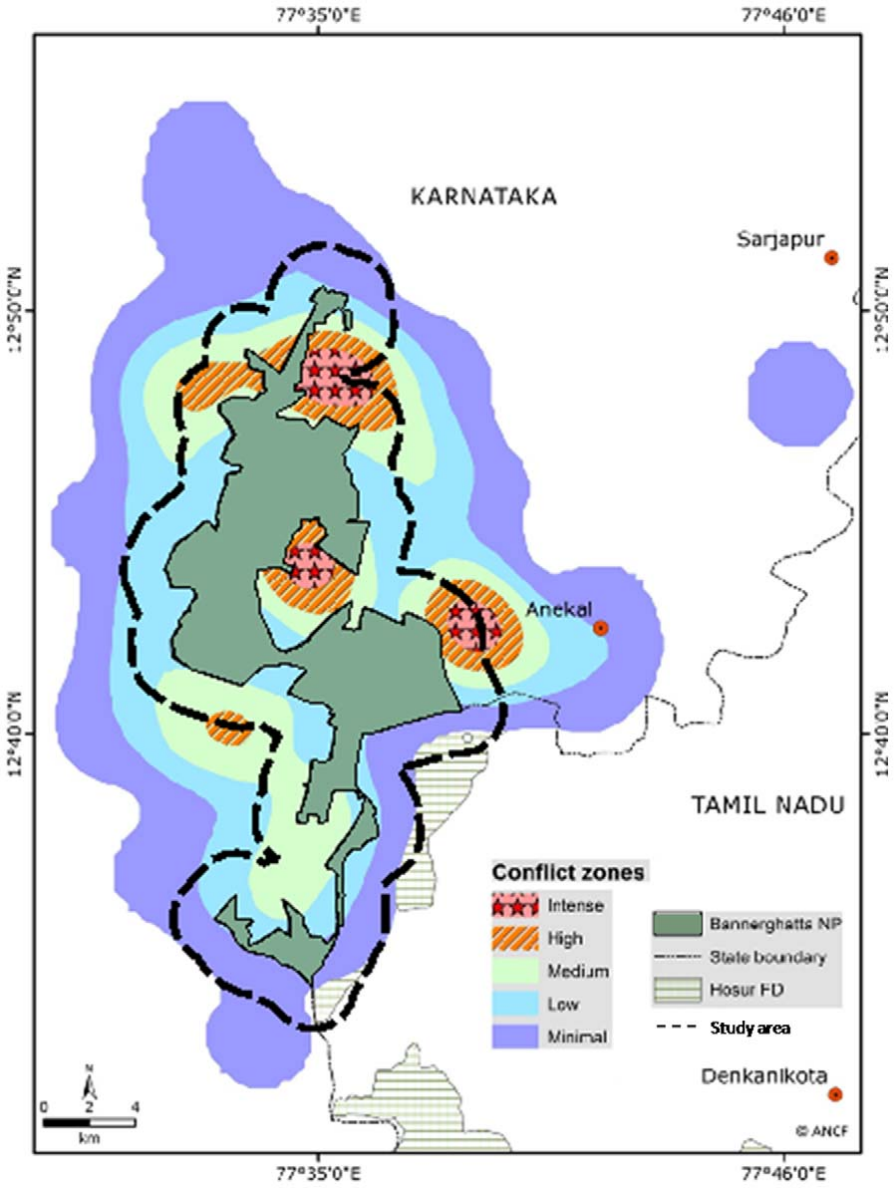
Map of the study area. The level of human-elephant conflict in the surrounding human-dominated landscape is shown.

This study was conducted in the Bannerghatta National Park and its surrounding human-dominated landscape (Figure 10), which forms a part of the Nilgiris – Eastern Ghats Elephant Reserve [34], [40]. It is one of the largest Elephant Reserves in India, with an area of approximately 11,000 km^2^. The Eastern Ghats Mountains also remains the last largest remaining scrub forest for elephants among its range countries and, along with another mountain chain, the Western Ghats, harbors the single largest contiguous Asian elephant population in the world, with an estimated number of about 9000 individuals [41].

The Park measures approximately 26 km in length and 0.30 to 5 km in width [34]. It has an area of about 104.27 km^2^ and a perimeter of 137 km. The terrain is highly undulating, with a mean altitude of 865 m above msl, and receives an average annual rainfall of 937 mm. The vegetation in the Park is predominantly deciduous to scrub woodland with riparian patches along the streams. Geographically, the Park is contiguous with larger patches of forests on its southeast and southwest.

The Park is surrounded on three sides by well-irrigated croplands and human habitations. There are five human settlements within the Park and around 117 settlements located within five km around the Park [34]. The high-density human population in and around the Park is largely comprised of subsistence farmers, livestock-grazers and manual laborers engaged in sand-mining and granite-quarrying. These communities depend on the forests for their non-timber forest produce, firewood and livestock-grazing. Amongst the farming communities, the majority are marginal farmers practicing subsistence agriculture with a handful of progressive farmers who grow commercial crops and have plantations. The major crops grown in this region are rice (*Oryza sativa*), finger millet (*Eleusine coracana*), castor (*Ricinus communis*), maize (*Zea mays*), banana (*Musa paradisiaca*), mango (*Mangifera indica*), sapota (*Achras zapota*), coconut (*Cocos nucifera*) and jackfruit (*Artocarpus heterophyllus*).

The number of incidents of crop depredation by elephants registered every year within and around the Park ranges from 470 to 1477 with a mean (± SE) of 900 (±151.33). The Forest Department records indicate that the conflict situation was at its peak in the years 2005–2006, recording 1477 cases and resulting in the Department spending INR 18,48,250 (USD 42,982) as compensation towards life and property damages alone. On an average, two people have been killed and two injured by wild elephants, and two elephants killed or poached in retaliation, each year since 1997 [40].

This study was conducted during the dry season [34], from December 2009 to May 2010, both within the Park and in the surrounding human-modified landscape. The study area, as a whole, could be classified into at least seven different land cover types, namely, dry deciduous forest (32.44%), scrub forest (11.44%), rocky outcrops and hills (5.22%), plantations (5.11%), cropfields (29.11%), sand and granite mines (8%) and human habitations (8.67%; see also Table 2).

### Study species and population

The Asian elephant has today been estimated to number about 35,000–50,000, spread across its range over thirteen Asian countries [23]. India has approximately 50% of the total population of wild Asian elephants (20,000 to 25,000), with southern India supporting around 10,000 elephants in the wild [42]. Owing largely to the pressures of hunting as well as habitat loss, fragmentation and degradation, the geographic range of the Asian elephant has, however, declined by more than 70% since the 1960's. The surviving populations are highly fragmented and the species is listed as Endangered in the *2011 IUCN Red List of Threatened Species* [43]; it is also included in the Appendix I of the Convention on International Trade in Endangered Species of Wild Fauna and Flora (CITES, 43) and in the Schedule 1 of the Wildlife Protection Act of India 1972 [44].

The study population, a sub-set of the larger Nilgiris – Eastern Ghats elephant population, is free ranging in the study area and occurs at a fairly high density of 0.90 elephants km^−2^ [45]. Elephants regularly move in and out of the study area, however, which could be expected given their migratory nature and the contiguity of the Park with neighboring elephant habitats to its southeast and southwest. The study population of 60 individuals (in 10 herds, 3 all-male groups and as 8 solitary males), which has been monitored since 2007 [34] for its demographic parameters, consists of 25 adult and 17 subadult elephants, their juveniles and calves.

### Habitat survey

In order to assess the effect of varying levels of resource availability and human-induced disturbance on the behavior of the study elephants, a habitat survey was carried out as a pre-requisite to quantify available resources and the intensity of human-induced disturbance. For this purpose, a total of 46 grids of 4 km^2^ each were overlaid on the study area using a topographic sheet (1∶250,000). The maximum average values of Normalized Difference Vegetation Index (NDVI) and Leaf Area Index (LAI) were extracted using MODIS data products for each of the 2×2 grids using the GIS software Quantum GIS [46] and Geographic Resources Analysis Support System [47]. The values of NDVI and LAI were used as surrogate measures of forage [48]–[51] and shade availability respectively. A sampling effort of four km (with eight straight line segments, each of 500 m) was invested in each grid with a total sampling effort of 184 km for habitat surveys to measure the number of water sources and human-induced disturbance, and evaluate the major land-use types. We stratified the study area into zones of low-, medium- and high levels of forage-, water- and shade availability, and human-induced disturbance, all the four variables being considered independently (Table 1). The values of NDVI, LAI, number of water holes per grid and the Human Disturbance Index (defined here as the encounter rate of human or human-associated disturbances×proportion of segments in which these disturbances were found) were respectively used as surrogate measures of these four variables and a quantile classification algorithm (Quantum GIS, version 1.4.0) was used to determine the cut-off ranges for each stratum. The distribution of the seven land-use patterns, mentioned above, across these resource availability and human disturbance strata was evaluated in order to examine the habitat characteristics of these strata (Table 2).

**Table 1:** Stratification of the study area into zones of low-, medium- and high levels of forage-, water- and shade availability, and human disturbance.

Forage	Range value of NDVI	Water	Range value of number of water holes per grid	Shade	Range value of LAI	Human disturbance	Range value of HDI
**Low**	4.29 to 5.11	**Low**	1 to 3	**Low**	5.30 to 7.10	**Low**	0 to 18
**Medium**	5.12 to 5.66	**Medium**	3 to 5	**Medium**	7.10 to 8.35	**Medium**	18 to 60.50
**High**	5.67 to 6.08	**High**	5 to 10	**High**	8.35 to 17.15	**High**	60.50 to 112

The stratification is on the basis of Normalized Difference Vegetation Index (NDVI), number of water holes per grid, Leaf Area Index (LAI) and Human Disturbance Index (HDI) respectively; The range value of each variable used for the classification has been shown.

**Table 2:** Distribution of land-use types observed in the study area across the zones of low-, medium- and high levels of forage-, water- and shade availability, and human disturbance.

	Percentage of zone under land-use type						
	DD	SC	RO	PL	CF	MI	HH
**Low forage**	14	14	6	4	41	7	13
**Medium forage**	45	12	6	7	17	6	7
**High forage**	44	7	3	5	25	11	5
**Low water**	32	5	6	7	29	8	15
**Medium water**	63	0	15	3	15	0	5
**High water**	54	0	4	14	19	0	9
**Low shade**	24	12	6	4	39	8	8
**Medium shade**	37	11	8	6	23	9	6
**High shade**	37	12	2	5	24	8	12
**Low human disturbance**	58	11	6	6	9	4	6
**Medium human disturbance**	18	12	6	3	38	15	8
**High human disturbance**	25	11	4	8	38	2	13

DD: Dry deciduous forest, SC: Scrub forest, RO: Rocky outcrops and hills, PL: Plantations, CF: Cropfields, MI: Sand and granite mines, HH: Human habitations.

### Demographic and behavioral sampling

This study is based on approximately 200 field-hours of demographic and behavioral observations (amounting to 663.25 elephant-hours) conducted on 60 individually identified elephants, when they were in herds, all-male groups or solitary. The difficult terrain and habitat, combined with tracking on foot, however, limited the hours of observations per day to a mean (± SE, range) of 3.03 (±0.45, 0.75–13.75) h. A photographic file with sighting number, location, gender, approximate age and distinguishing morphological features was thus created for each individual elephant. The age of each individual was estimated from its physical characteristics and comparative measures of shoulder height [34], [52]. The study elephants were classified into three group types, namely, solitary (single male elephant), all-male group (AMG, a coalition of male elephants comprising at least one adult and one or more subadult males, mean ± SE group size of 2.50±0.50, range 2 to 3) and herd (one or more family units with mean ± SE group size of 6.60±2.10, range 3 to 12) on the basis of their group size and structure. They were further grouped into four age classes, namely, adult (>15 years, mean ± SE of 34.22±1.52 years, range 20 to 50 years), subadult (range of 5 to 15 years, mean ± SE of 11.79±0.53 years), juvenile (1 to 5 years, mean ± SE of 3.75±0.28 years, range 2 to 5 years) and calf (<1 year). The adult male to female ratio was 1∶2.57 and the adult female to calf ratio 1∶ 0.28.

The location of each identified elephant, whether solitary or in a herd or all-male group, was obtained once every 15 min using an e-trex Global Positioning System (GPS; Garmin Corp, Kansas, USA). Each recorded point was then assigned to the grid in which the elephant was observed. This data was further processed to class the grid into one of the 12 strata described above on the basis of resource availability and human disturbance (Table 2). The probability of individual elephants occurring in one of the 12 strata as a function of their gender, age and group type was then assessed. Observed Residence Time (ORT) has been defined as the total time spent by elephants in grids assigned to a particular stratum of resource availability or human disturbance. Expected Residence Time (ERT) was calculated by multiplying the sample size obtained from ORT with the area under each stratum.

Behavioral states including feeding, resting, moving, bathing/drinking and social interactions were recorded by instantaneous scan sampling of all visible individuals, whether solitary or in an association, at 15-min intervals [53]. Sampling was conducted during three sessions of three-hour duration each (Session 1∶ 0700 to 1000 h; Session 2∶ 1100 to 1400 h; Session 3∶ 1500 to 1800 h) within each observation day. The scan data were used to calculate the time-activity budget of the study elephants and also to examine the effect of resource availability and human disturbance on their behavioral decision-making. Extensive *ad libitum* observations were carried out during a fourth nocturnal session, which extended from 1900 to 0600 h. These data were largely used to estimate elephant occurrence and residence time in the different strata.

The study elephants were classified into three group types described above, on the basis of their group size and structure. These association data were recorded at first- and last sighting for each of the four sessions of observations (Session 1∶ 0700 to 1000 h; Session 2∶ 1100 to 1400 h; Session 3∶ 1500 to 1800 h and Session 4∶ 1900 to 0600 h) within each observation day. The frequency with which these group types were sighted in the 12 different strata during each session was then analyzed.

Inter-Individual Distance and the frequency of audible vocalizations were observed across all four sampling sessions to assess the effect of human disturbance on the social interactions of the study elephants including their auditory communication. For Inter-Individual Distance, the distance between all visible individuals of the focal herd was measured once every 60 min using scan sampling. The frequency of auditory vocalizations displayed by the elephants was recorded either during focal herd or group sampling or focal animal sampling (for solitary individuals), conducted for a period of 10 min each, following each instantaneous scan described above. Each focal group or animal sampling was followed by a 5-min rest period. Each event of vocalization was assigned to the grid in which the vocalization was heard and later classed into one of the three human disturbance zones. The movement rate of elephants was measured in terms of the total distance travelled by them within each stratum of human disturbance. The GPS locations of the elephants were also used to obtain the movement rate of solitary individuals, herds or all-male groups, measured in terms of the Euclidean distance covered in unit time using the package Adehabitat in R, version 2.9.2 [54].

### Statistical analysis

In order to examine decision-making by individual elephants, when solitary or in association with conspecific individuals, we constructed recursive partitioning classification trees. This method estimates a regression relationship by binary recursive partitioning in a conditional inference framework [55] in the following manner. The global null hypothesis of independence between any of the input variables (the gender, age, group type and individual identity of an elephant) and the response variable (resource availability or human disturbance) was first tested and the hypothesis accepted if it could not be rejected. If the null hypothesis could be rejected, the input variable with the strongest association to the response variable was selected and their association measured by a p-value corresponding to a test for the partial null hypothesis of a single input variable and the response variable. A binary split was then implemented in the selected input variable. The above steps were repeated recursively until a statistically significant binary partitioning of the input variable could not be derived further. The implementation utilized a unified framework for conditional inference or permutation tests, developed by Strasser and Weber (1999). The criterion for the first binary spilt in the input variable was based on multiplicity-adjusted Monte-Carlo-simulated p-values. A split was implemented at the node when the simulated p-value was smaller than 0.05. This statistical approach ensured that the right-sized tree was grown and no form of pruning or cross-validation was required.

We assessed differences in the behavioral responses of elephants (probabilities of occurrence in particular strata, time-activity budgets, social interactions and demographic structure) to varying levels of resource availability and human-induced disturbance as a function of their gender, age and group type using G-tests of independence and goodness of fit. The former test was also used, where appropriate, as a post-hoc procedure to assess the statistical significance of the recursive partitioning classification trees obtained by exploratory analysis. Data on the display of IID and AV as a function of human-induced disturbance were analyzed using the Wilcoxon rank sum test with continuity correction and G-test of goodness of fit respectively [53].

## Supporting Information

Figure S1 **Classification trees showing the partitioning of elephant group size in the different strata.** L: low-; M: medium- and H: high (a) water- and (b) shade availability. The y-axis of each graph indicates the proportion of groups observed in the different strata.
(TIF)

Figure S2 **Classification trees for residence time of female and male elephants in the different strata.** The trees depict the influence of (a) shade availability and (b) human disturbance. The y-axis of each graph indicates the proportion of groups observed in the different strata.
(TIF)

Figure S3 **Classification trees for residence time of adult and subadult male elephants in the different strata.** The trees depict the influence of (a) shade availability and (b) human disturbance. The y-axis of each graph indicates the proportion of groups observed in the different strata.
(TIF)

Figure S4 **Classification trees for residence time of adult and subadult female elephants in the different strata.** (a) Forage-, (b) water- and (c) shade availability and (d) human disturbance. The y-axis of each graph indicates the proportion of groups observed in the different strata.
(TIF)

Figure S5 **Classification trees of the probability of individual adult and subadult male elephants occurring in the different strata.** (a) water- and (b) shade availability. The y-axis of each graph indicates the proportion of groups observed in the different strata.
(TIF)

Figure S6 **Classification trees of the probability of an individual herd occurring in the different strata.** L: low-; M: medium- and H: high (a) water- and (b) shade availability. The y-axis of each graph indicates the proportion of groups observed in the different strata.
(TIF)
