# Correction: Usual Populations, Unusual Individuals: Insights into the Behavior and Management of Asian Elephants in Fragmented Landscapes

**DOI:** 10.1371/annotation/0674886e-ce0a-4432-8433-fc16c8e7e925

**Published:** 2012-10-23

**Authors:** Nishant M. Srinivasaiah, Vijay D. Anand, Srinivas Vaidyanathan, Anindya Sinha

There is an error in affiliation 1 for author Nishant M. Srinivasaiah. Affiliation 1 should be: Postgraduate Program in Wildlife Biology and Conservation, Wildlife Conservation Society - India Program, National Centre for Biological Sciences, Tata Institute of Fundamental Research, GKVK Campus, Bangalore, India 

