# Supplementary figures and images for: Usual Populations, Unusual Individuals: Insights into the Behavior and Management of Asian Elephants in Fragmented Landscapes
Source: PLoS One. 2012 Aug 15;7(8):e42571. doi: 10.1371/journal.pone.0042571 (PMC3419726; doi:10.1371/journal.pone.0042571)

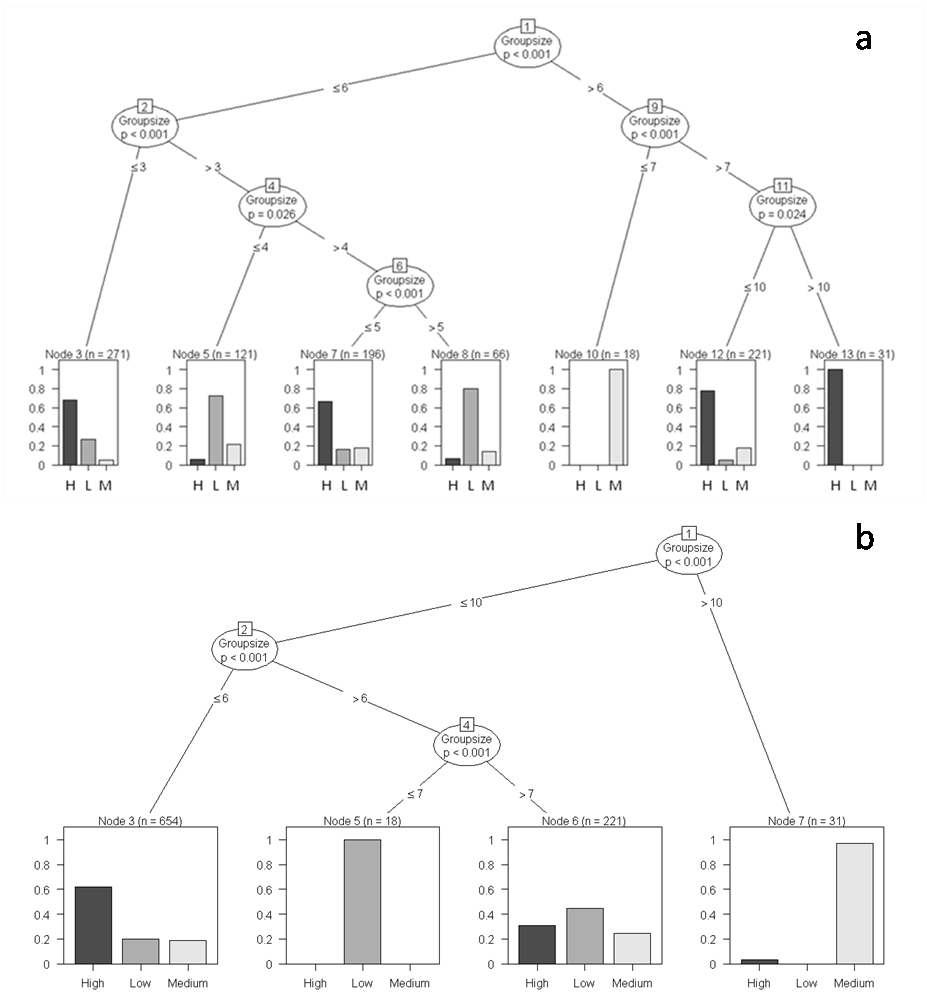

Supplement: Figure S1 — Classification trees showing the partitioning of elephant group size in the different strata. L: low-; M: medium- and H: high (a) water- and (b) shade availability. The y-axis of each graph indicates the proportion of groups observed in the different strata. (TIF) [file pone.0042571.s001.tif]

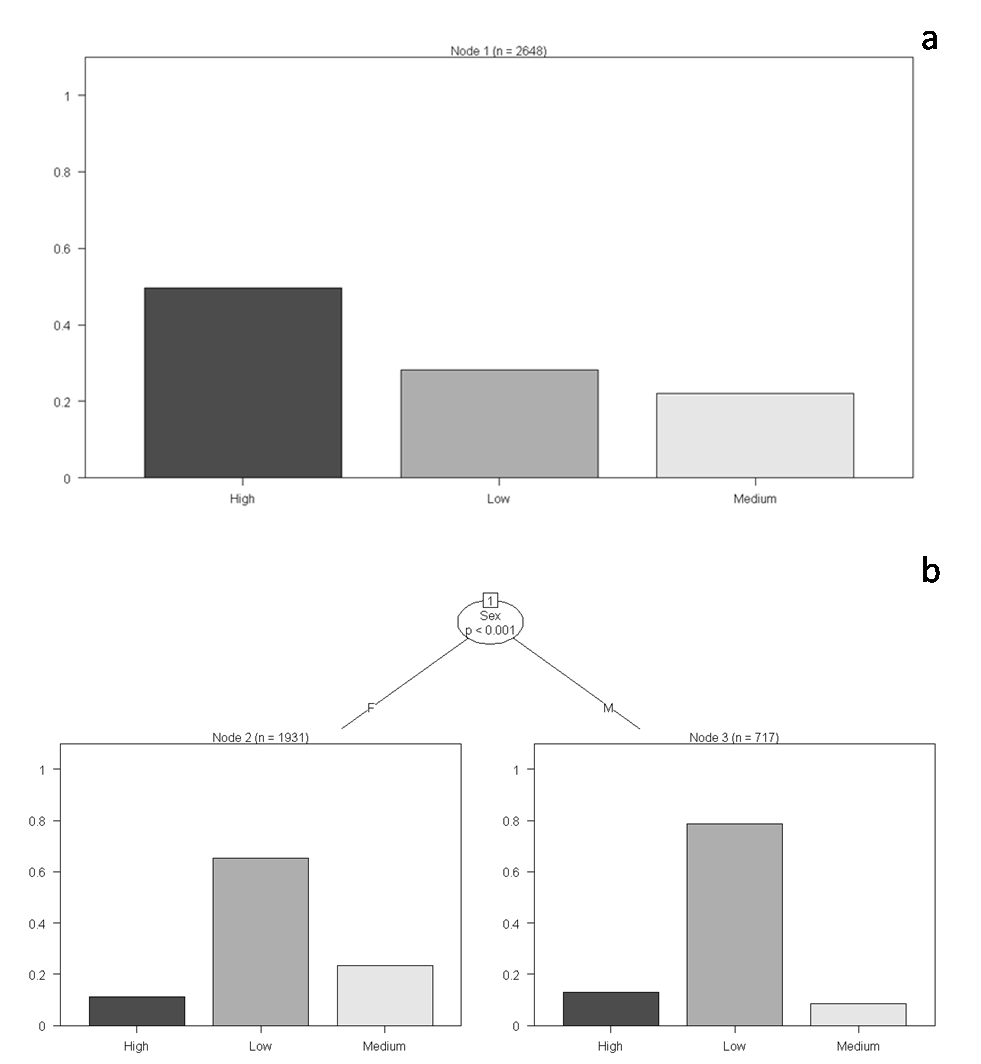

Supplement: Figure S2 — Classification trees for residence time of female and male elephants in the different strata. The trees depict the influence of (a) shade availability and (b) human disturbance. The y-axis of each graph indicates the proportion of groups observed in the different strata. (TIF) [file pone.0042571.s002.tif]

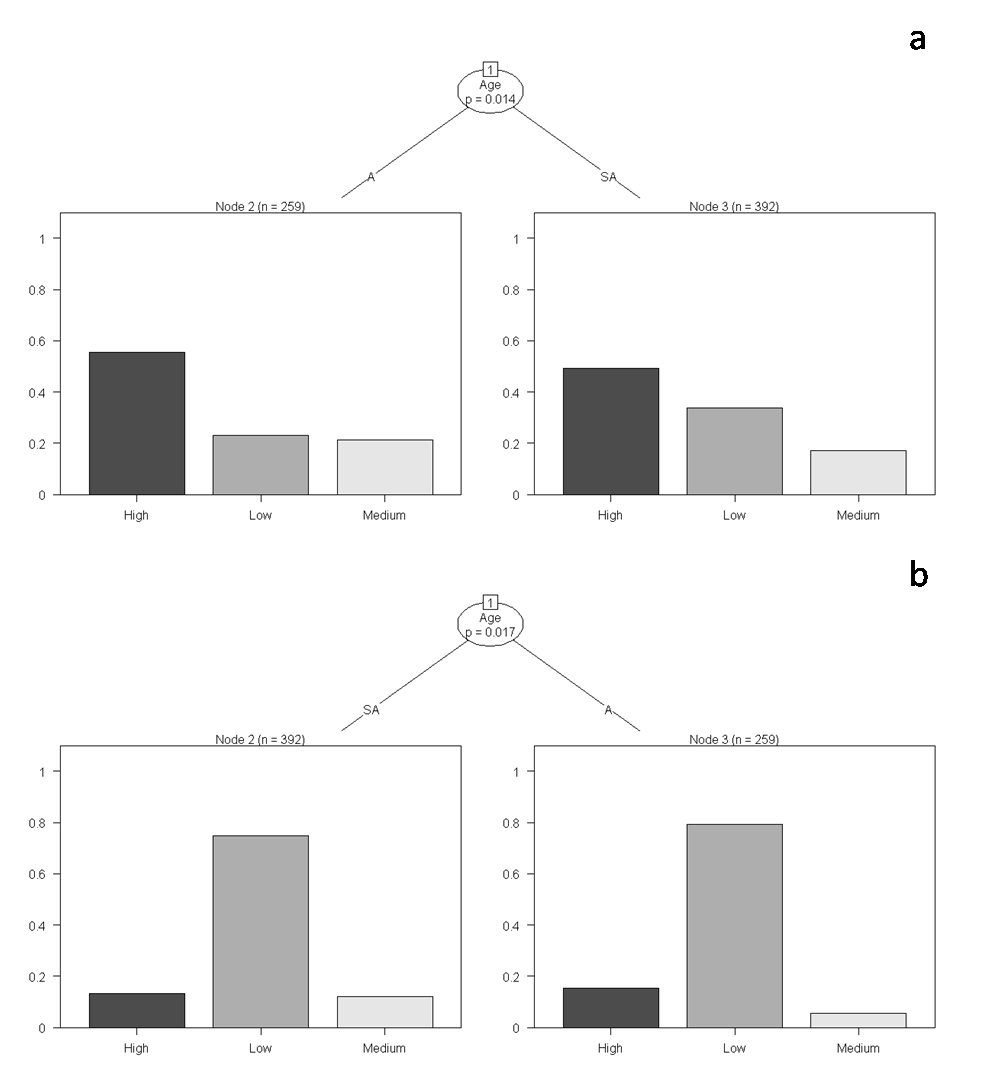

Supplement: Figure S3 — Classification trees for residence time of adult and subadult male elephants in the different strata. The trees depict the influence of (a) shade availability and (b) human disturbance. The y-axis of each graph indicates the proportion of groups observed in the different strata. (TIF) [file pone.0042571.s003.tif]

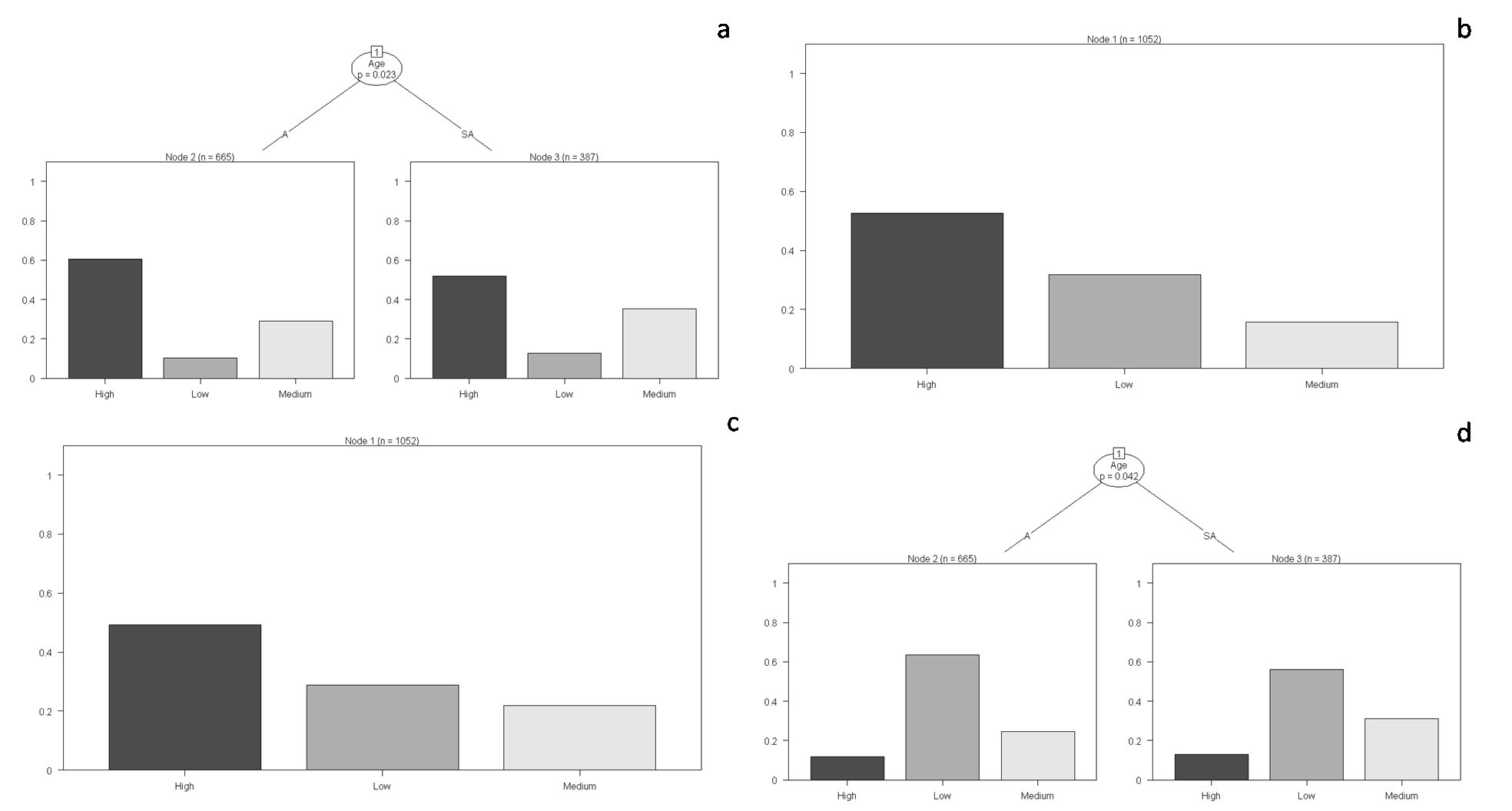

Supplement: Figure S4 — Classification trees for residence time of adult and subadult female elephants in the different strata. (a) Forage-, (b) water- and (c) shade availability and (d) human disturbance. The y-axis of each graph indicates the proportion of groups observed in the different strata. (TIF) [file pone.0042571.s004.tif]

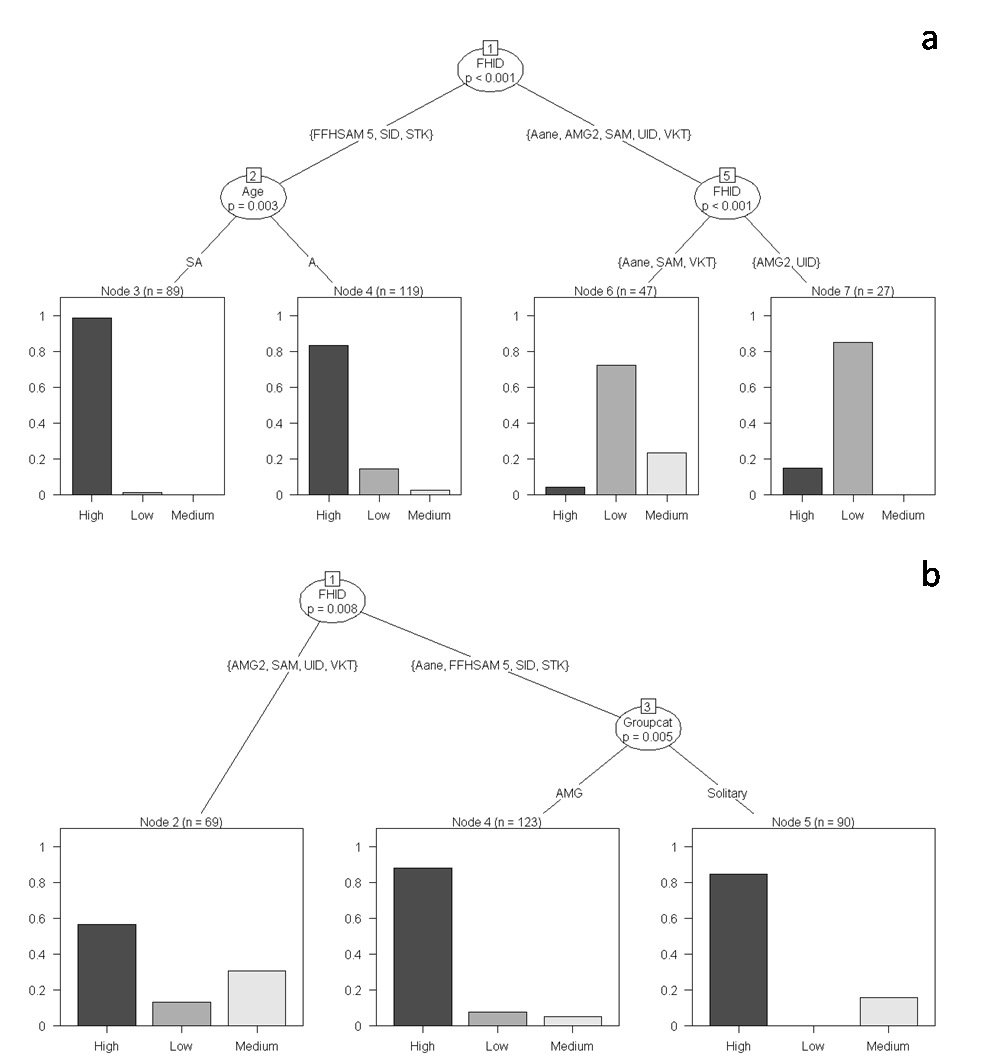

Supplement: Figure S5 — Classification trees of the probability of individual adult and subadult male elephants occurring in the different strata. (a) water- and (b) shade availability. The y-axis of each graph indicates the proportion of groups observed in the different strata. (TIF) [file pone.0042571.s005.tif]

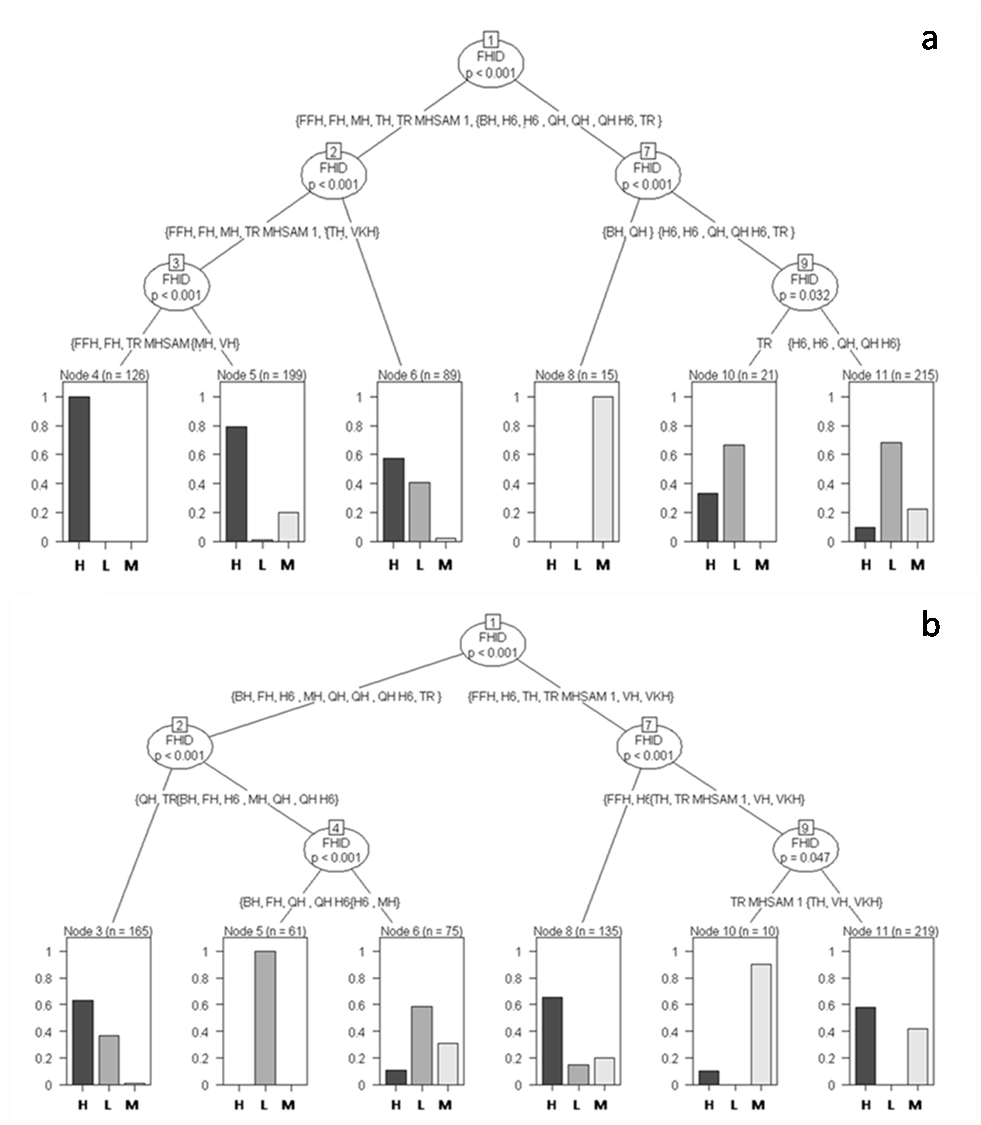

Supplement: Figure S6 — Classification trees of the probability of an individual herd occurring in the different strata. L: low-; M: medium- and H: high (a) water- and (b) shade availability. The y-axis of each graph indicates the proportion of groups observed in the different strata. (TIF) [file pone.0042571.s006.tif]
